# Impact of sex, race, and social determinants of health on neonatal outcomes

**DOI:** 10.3389/fped.2024.1377195

**Published:** 2024-04-09

**Authors:** Pradeep Alur, Ira Holla, Naveed Hussain

**Affiliations:** ^1^ Penn State College of Medicine, Hampden Medical Center, Enola, PA, United States; ^2^ Department of Pediatrics, University of Mississippi Medical Center, Jackson, MS, United States; ^3^ Department of Pediatrics, Connecticut Children’s, Hartford, CT, United States

**Keywords:** sex differences, newborns, racial differences, social determinants of health, outcomes

## Abstract

Despite the global improvements in neonatal outcomes, mortality and morbidity rates among preterm infants are still unacceptably high. Therefore, it is crucial to thoroughly analyze the factors that affect these outcomes, including sex, race, and social determinants of health. By comprehending the influence of these factors, we can work towards reducing their impact and enhancing the quality of neonatal care. This review will summarize the available evidence on sex differences, racial differences, and social determinants of health related to neonates. This review will discuss sex differences in neonatal outcomes in part I and racial differences with social determinants of health in part II. Research has shown that sex differences begin to manifest in the early part of the pregnancy. Hence, we will explore this topic under two main categories: (1) Antenatal and (2) Postnatal sex differences. We will also discuss long-term outcome differences wherever the evidence is available. Multiple factors determine health outcomes during pregnancy and the newborn period. Apart from the genetic, biological, and sex-based differences that influence fetal and neonatal outcomes, racial and social factors influence the health and well-being of developing humans. Race categorizes humans based on shared physical or social qualities into groups generally considered distinct within a given society. Social determinants of health (SDOH) are the non-medical factors that influence health outcomes. These factors can include a person&apos;s living conditions, access to healthy food, education, employment status, income level, and social support. Understanding these factors is essential in developing strategies to improve overall health outcomes in communities.

Despite the global improvements in neonatal outcomes, mortality and morbidity rates among preterm infants are still unacceptably high. Therefore, it is crucial to thoroughly analyze the factors that affect these outcomes, including sex, race, and social determinants of health. By comprehending the influence of these factors, we can work towards reducing their impact and enhancing the quality of neonatal care. This review will summarize the available evidence on sex differences, racial differences, and social determinants of health related to neonates. This review will discuss sex differences in neonatal outcomes in part I and racial differences with social determinants of health in part II.

Method of Review: The authors reviewed published literature on sex-based differences related to the specific items of interest discussed in the manuscript. They did a comprehensive review and synthesis of available information. The authors' consensus on the inclusion and exclusion of available information pertinent to the focus of this non-structured review was based on this.

## Part I. Sex Differences in Neonatal Outcomes

It is widely acknowledged that there are differences in outcomes between male and female neonates. However, research has shown that sex differences begin to manifest in the early part of the pregnancy. Hence, we will explore this topic under two main categories: (1) Antenatal and (2) Postnatal sex differences. We will also discuss long-term outcome differences wherever the evidence is available.

### Antenatal outcomes

Considerable information on sex differences in human pregnancy is accumulating rapidly. This review cannot present all the available evidence. However, we will primarily focus on how fetal sex can affect maternal outcomes and how maternal conditions and toxins can affect the sexes differently.

#### Effect of fetal sex on maternal outcomes

The genetic or gonadal sex of the fetus has several ramifications that extend beyond the complications of the pregnancy. The sex of the fetus not only determines its own outcome but may impact the mother's as well. A few studies have found that pregnant females carrying a male fetus had a higher risk of developing gestational diabetes mellitus (GDM) and higher postprandial glycemia compared to females carrying a female fetus [odds ratio: 1.39 (95% CI: 1.01–1.90)] (1, 2). Similarly, in a study of 108,995 deliveries in Israel, investigators in their retrospective analysis found that pregnant females with a male fetus had a higher incidence of GDM than if carrying a female fetus [unadjusted OR: 1.1 (95% CI: 1.01–1.12)] (2) Thus, the fetus can influence maternal metabolism. Preterm pre-eclampsia was found to be more prevalent in females carrying female fetuses than males (3). Others have reported slight male preponderance in term and post-term pre-eclampsia. Some speculate that pregnancies with a male embryo are susceptible to poor placentation, whereby the pregnancies with a male embryo that are prone to developing pre-eclampsia due to diminished placentation may have already been aborted during the first trimester. The male fetuses that survive the period of placentation are, therefore, expected to represent a comparatively healthy group of fetuses, leading to a higher incidence of pre-eclampsia among females (4). On similar lines, preterm birth, for which placental insufficiency is one of the important causes, is also common with male fetuses.

It is argued that conceiving more sons is energy expensive for mothers than having daughters. Observational studies in Scandinavia during the pre-industrial era showed that maternal longevity did not correlate with the total number of children. However, giving birth to sons compared to daughters significantly shortened mothers' lifespan (5). Similarly, mothers reported more health issues in post-reproductive age if they had more sons than daughters. Each son increased the odds of health issues by 11% (6).

#### Basis for antenatal sex differences

Sex chromosomes and sex hormones play different roles at various stages of gestation, contributing to sex differences.

Early human embryo studies suggest X chromosome inactivation occurs in female embryos around 12 days to a month post-fertilization. However, X-linked transcripts are expressed 2-fold higher in females before X chromosome inactivation. Some studies suggest that differences in preimplantation growth may be because of the X chromosome. Sex differences in fetal development during the first trimester are most likely caused by the expression of genes on the sex chromosomes or other effects related to the sex chromosomes. This is because the production of sex steroid hormones in the fetus begins later in the first trimester (7).

The fetal testis secretion of testosterone is essential for male gonadal tract formation and defeminization and masculinization of male fetal brains. The testosterone secretion from fetal Leydig cells begins around 8–10 weeks of gestation and peaks at post-pubertal levels by 16 weeks (8). Male fetuses exhibit higher testosterone levels between 12 and 16 weeks of gestation, contributing to sex-specific phenotypic differences such as rapid growth of external genitalia (9).

Both sexes are exposed to estrogens throughout the pregnancy, and deficiency of estrogens was not found to affect fetal growth. No sex differences were noted with prenatal levels of estrone, estradiol, Estriol, and dehydroepiandrosterone (9, 10). The hypophyseal portal system is well developed by 18 weeks and begins releasing hormones to the anterior pituitary (11). However, the human placentas can synthesize androgens and testosterone by the first trimester, and the early sex hormone effect is likely from the placental origin (12). Studies have shown that placental biomarkers such as placental growth factor and plasminogen activator inhibitor were significantly increased in females carrying female fetuses, suggesting a more vascularized placenta throughout pregnancy in uncomplicated pregnancies (7).

#### Fetal growth

The differences in fetal growth are described with higher growth rates in male fetuses (7). The differences in growth become marked by the second trimester. Please refer to a more extensive review on sex differences in prenatal growth published previously (13).

Effect of twin gestation: According to research, male/male vs. female/female twin gestation and male/female twin gestations can lead to different outcomes. A registry-based cross-sectional multicenter study conducted in Japan analyzed 23,804 females with dichorionic diamniotic (DD) twins and 14,149 females with monochorionic diamniotic (MD) twins (14). According to the study, females who had male/male DD twins were at a higher risk of preterm birth (adjusted risk ratio [aRR]: 1.07, 95% confidence interval [CI]: 1.03–1.10) and a lower risk of preeclampsia (aRR: 0.74, 95% CI: 0.62–0.88) when compared to females who had female/female DD twins. Similarly, females with male/male MD twins had a higher risk of preterm birth (aRR: 1.06, 95% CI: 1.04–1.09) than females with female/female MD twins. Additionally, male small for gestation (SGA) risk was significantly higher among male/male twins than male/female DD twins. Among MD twins, the risks of SGA and fetal death were significantly higher in male/male fetuses. There was a marked difference between fetal males and females in their growth trajectory for fetal head measurements. Hence, using sex-specific fetal growth charts is essential for accurate second-trimester dating.

#### Sexual dimorphic effects of maternal conditions

Adverse maternal conditions affect male and female fetuses differently. Overall, female fetuses appear to be favored to survive the suboptimal intrauterine conditions.

(a) Asthma: If a woman experiences mild asthma during pregnancy, it may affect the growth of her female fetus but not enough to cause intrauterine growth restriction (IUGR). However, male fetuses can develop strategies that help them grow normally, even in adverse intrauterine conditions. Nevertheless, this makes them vulnerable to potential health risks in case of a second stressful event, such as an acute asthma attack. On the other hand, females tend to adapt to a poor intrauterine environment caused by chronic maternal asthma by reducing their growth. As a result, they become smaller but not IUGR. This adaptation helps them survive further compromises in the intrauterine environment, such as lack of oxygen or nutrition, as the pregnancy progresses (15). Similar sex-specific growth strategies are reported with mild pre-eclampsia as well.
(b) Immune response: Sex-specific fetal immune responses have been observed. The Alabama Preterm Birth Study noted that male infants born 23–32 weeks gestation were likelier to have positive placental cultures than female infants [63.4% vs. 51.8%, *p* = .01, with an odds ratio: 1.5, (95% CI): 1.0–2.4] (16). The authors noted higher rates of chronic decidual inflammation in the placentas in male infants. Similarly, Ghidini et al. reported chronic decidual inflammation in the placentas with male infants at the interface between interstitial trophoblast and maternal decidua (17). They speculated that maternal immune response varies with the sex of the fetus. Investigators found differences in placental gene expression and antibody transfer in response to maternal SARS-CoV-2 infection based on sex. Maternal SARS-CoV-2 infection has been found to result in reduced levels of SARS-CoV-2-specific IgG in mothers, especially when the fetus is male. In male fetuses exposed to SARS-CoV-2, there is a decrease in the transfer of SARS-CoV-2-specific antibodies from the placenta. Although placental Fc receptors are up-regulated, IgG levels against SARS-CoV-2 antigens are significantly lower in cord blood of male fetuses than in maternal plasma. By contrast, pregnancies with female fetuses exhibit no significant difference in IgG titers between cord blood and maternal plasma. This may be attributed to the male fetus's inability to overcome the reduced maternal titers. These findings provide insights into the altered maternal-placental-fetal immune crosstalk in the presence of viral infection during pregnancy, with fetal sex playing a crucial role in modifying maternal humoral responses (18). Animal studies have reported that group B strep-induced immune signaling involved significantly higher cytokine levels in male maternofetal tissues than in females (19). However, investigators in South Africa looked at the sex-specific differences in in-utero HIV infection and reported that female fetuses had 1.5–2-fold increased susceptibility to intrauterine HIV infection (20). Maternal stress, in general, causes sexually dimorphic responses in the offspring. Males tend to have a higher incidence of autism spectrum disorders, whereas females experience more depression. A prospectively analyzed data from more than 15,000 pregnancies followed over 40 years showed that maternal bacterial infections were associated with a higher incidence of psychosis in males than in females (21). It is suggested that the observed higher levels of proinflammatory cytokines in male fetuses compared to female fetuses in response to bacterial endotoxins may explain why male fetuses are prone to psychosis later in life, given the fact that proinflammatory cytokines have long been implicated in schizophrenia and other psychotic disorders (21). However, the specific mechanism for why males are susceptible is unclear as human evidence is still evolving.

It has been shown that exposure to proinflammatory cytokines *in utero* was significantly associated with male and female differences in brain activity and connectivity measured 45 years later using negative, stressful stimuli and brain MRI responses (22). The study results indicated that lower levels of maternal TNF-α were associated with increased hypothalamic activity in response to negative stimuli in male and female offspring. Typically, the hippocampus provides negative feedback to the hypothalamus in response to negative, stressful stimuli, enabling the hypothalamic-pituitary axis to inhibit corticotropin-releasing hormone release and regulate arousal due to stress. With higher TNF-α (proinflammatory): IL-10 (anti-inflammatory) exposure, males had lower connectivity between the hypothalamus and hippocampus and thus less ability to inhibit the higher activity of the hypothalamus by the hippocampus. Females who were exposed to higher levels of TNF-α:IL-10 showed increased connectivity between the hippocampus and hypothalamus. However, they also had lower hippocampal activation, which reduced their ability to down-regulate hypothalamic arousal and potentially corticotropin-releasing hormone release. It is noteworthy that the dysregulation pattern differed in male and female offspring. Females had higher hippocampal activity with elevated prenatal IL-6 levels. The data suggests that male fetuses bias the maternal immune response toward a Th1 inflammatory response, while female fetuses can trigger a more regulatory Th2-like maternal immune response (12, 23).

#### Sexual dimorphic effects of environmental toxins

Antenatal exposure to environmental toxins also has differential effects based on sex. Emerging evidence suggests that female fetuses are more tolerant of exposure to intrauterine toxins. In a prospective study conducted in Cincinnati, male infants exhibited a more significant neurobehavioral deficit at six months in response to fetal exposure to lead, as assessed by maternal blood (24). In another study, prenatal lead exposure was linked with lower cognitive function in males (Spearman correlation coefficient = −0.239, *p* = 0.0007), but not in females (25). Methyl mercury exposure antenatally also showed sex-specific effects. Males were significantly more affected than females in childhood (26, 27). Males were noted to manifest more ADHD-related behaviors than females following antenatal mercury exposure (28). Many of these substances are considered endocrine disruptors causing sex-specific effects.

A prospective study from China found that maternal arsenic levels were associated with specific adverse birth outcomes only in females during the third trimester. Doubling of 3rd-trimester arsenic levels was linked to a decrease of 37.66 g (95% CI: −71.57, −3.75) in birth weight, a decrease of 0.19 cm (95% CI: −0.32, −0.06) in birth length, and a 34% increase in the risk of SGA (95% CI: 1.03, 1.73) in females (29).

Following antenatal exposure to opioids such as methadone and buprenorphine, males manifested more severe symptoms than females postnatally (30, 31). Interesting observations were noted with maternal smoking. Smoking ½ packet per day resulted in smaller weight and head circumferences in males than in females (32). In a study involving 454 infants, antenatal smoking exposure specifically was associated with lower levels of social-approach-related behavior, gross motor movement, reactivity, and attention in male infants (33). These effects may be related to differential activation of the hypothalamic–pituitary–adrenal (HPA) axis, as evidenced by lower salivary cortisol levels in males exposed to maternal tobacco than in the control males. Such an effect was not noted in females (34).

### Postnatal outcomes

Evolving evidence suggests sex differences in postnatal complications among preterm and term infants (Table 1). We provide a summary of the current evidence based on the affected systems.

**Table 1:** Summary of sex-differences in postnatal complications.

Postnatal complications	Outcomes in males vs. females
Intraventricular Hemorrhage	Higher in preterm males across birth weight categories (RR: 1.271, 95% CI: 1.207–1.338, *p* < 0.001) (35, 36). Significant reduction with prophylactic indomethacin in incidence and severity of IVH in male infants as compared to saline controls (R = 0.34, *p* = 0.007), but no change in IVH rate or severity in females (37).
Hypoxic Ischemic Encephalopathy	Significant but modest male predominance in a global metanalysis of incidence of Intrapartum HIE (58.7%, 95% CI: 55.6–61.8) (38). Animal studies have shown that males tend to have worse outcomes associated with HIE than females (39). Post hoc analysis of the data from the Hypothermia trial (40) failed to show a sexual dimorphism in the treatment effect of hypothermia in infants with moderate or severe neonatal encephalopathy (41).
Neonatal Abstinence Syndrome	Male infants were more likely to be diagnosed with NAS than females [adjusted odds ratio: 1.18 (95% confidence interval: 1.05–1.33)] and more likely to have NAS requiring treatment [adjusted odds ratio: 1.24 (95% confidence interval: 1.0401.47)] (42).
Retinopathy of Prematurity	Males are more likely to be screened for Retinopathy of Prematurity (ROP), and a higher percentage of male infants are treated for ROP as compared to female infants (55%, 95% CI: 52–58%) (43). Males are also at higher risk having severe ROP (RR: 1.14, 95% CI: 1.07–1.22) (36, 44), suggesting that males are at higher risk of progression of ROP.
Neurodevelopmental Impairment	Male sex has been identified in multiple studies as an independent risk factor for poor neurodevelopmental outcomes, including cerebral palsy (45–47), with Chounti et al. finding that males have a 30% higher incidence (95% CI: 11%–53% *p* = 0.002) of CP.
Respiratory Distress Syndrome	Male infants born preterm are at increased risk of RDS as compared to females (RR = 1.090, 95% CI: 1.042–1.140, *p* < 0.001) (36), as well as increased severity of RDS (48) with more males born <29 weeks gestational age requiring mechanical ventilation and surfactant therapy in the first 6 h of life as compared to females (60.8% males vs. 46.2% females, *p* = 0.026).
Bronchopulmonary Dysplasia	BPD was found to be more common in males than in females among infants born at 24–27 weeks (*p* < 0.001) but not significantly different among males and females born at 22–23 weeks (49). In infants with established BPD, there was no sex-related difference in rates of mechanical ventilation at 36 weeks (males 5.23%, females 3.6%), with OR = 1 for death or tracheostomy for male-to-female infants (95% CI: 0.7–1.5) (50).
Patent Ductus Arteriosus	No significant sex-related difference in the risk of developing any PDA (37 studies, RR: 1.03, 95% CI: 0.97–1.08), risk of hemodynamically significant PDA (81 studies, RR: 1.00, 95% CI: 0.97–1.02), or rate of response to pharmacological treatment of PDA (45 studies, RR: 1.01, 95% CI: 0.98–1.04) (51).
Hypotension	In infants <1,000 grams birth weight, males were at higher risk of requiring inotropic support in the first 4 days of life as compared to females (67.1% vs. 50.6%, *p* < 0.05), had lower mean arterial blood pressure in the first 12–24 h of life (25 vs. 28, *p* < 0.05), and were more likely to develop “resistant hypotension” defined as the need for dobutamine or hydrocortisone in addition to volume and dopamine to treat hypotension (25.9 vs. 11.5, *p* < 0.05) (48).
Sepsis	Male infants were at a significantly greater risk of late-onset sepsis as compared to female infants (R = 1.051, 95% CI: 1.026–1.077, *p* < 0.001) (36). Male sex was an independent risk factor for early onset sepsis (RR = 2.7, 95% CI: 2–5) (52). Estradiol inhibits the stimulatory effect of LPS (Lipopolysaccharide) on the Hypothalamic-Pituitary Axis (HPA) in adult females (53), and Estriol sensitizes Kupffer cells to LPS (54), both of which are theorized to be the explanation for the sex-based difference in risk of neonatal sepsis.
Necrotizing enterocolitis	Among infants born between 22 and 29 weeks gestational age, males had a significantly higher rate of NEC than females (10.1% vs. 8.4%, AOR = 1.2, 95% CI: 1.17–1.24) (55). Among infants with a diagnosis of NEC, female sex was associated with a 3-fold increase in risk of mortality (56).

#### Central nervous system

##### Short term

One of the significant risk factors for poor long-term neurodevelopmental outcomes is brain injury in the neonatal period in the form of Neonatal Encephalopathy, Intraventricular Hemorrhage, or white matter injury, to name a few.

##### Intra-ventricular hemorrhage (IVH)

While the percentage of infants with Severe Intraventricular Hemorrhage (sIVH, grade III and IV IVH) has shown a decreasing trend over time (57), a recent review showed a worldwide incidence of 6%–10% among infants born at less than 28 weeks gestational age (58). Incidence and severity of intraventricular hemorrhage are known to be higher in preterm males across birth weight categories (RR: 1.271, 95% CI: 1.207–1.338, *p* < 0.001) (35, 36). Multiple theories have been proposed to explain this sexual dimorphism, one of which is a finding of increased cerebral blood flow and increased cerebral vasoreactivity to carbon dioxide levels in males (59, 60). Another proposed mechanism of increased male susceptibility to brain injury could be a difference in the immune response to injury between males and females (61). Fine et al. (62) noted a sex difference in the inflammatory response, with males demonstrating a heightened response to endotoxin stimulation. The X chromosome plays a crucial role in immune modulation by encoding several immune-related genes that may confer a female advantage. The Interleukin 1 Receptor Associated Kinase 1 (IRAK1) gene and Toll-like receptor 4 (TLR4) gene are a few examples that are expressed differently in females as compared to males and are critical in regulating immune responses to pathogens (63, 64). Animal studies have also demonstrated increased susceptibility of the male preterm brain to injury secondary to differences in in-utero intracerebral sex hormone levels (65). The degree of response to therapies and methods of preventing IVH has also been noted to vary based on sex. Ment et al. (37) studied the effect of prophylactic indomethacin in 432 very low birth weight infants and found a significant reduction in incidence and severity of IVH in male infants as compared to saline controls (RR = 0.34, *p* = 0.007) but found no change in IVH rate or severity in females. However, a secondary analysis of the 558 infants enrolled in the Trial of Indomethacin in Preterm Infants (TIPP) (66) found only a weak differential in response by sex (*p* = 0.29) (67). Antenatal steroids were also noted to produce a more significant reduction in the incidence of IVH in females as compared to males. However, both sexes benefited significantly from antenatal steroid exposure (incidence of IVH 10.9% vs. 13.9%, *p* < 0.001) (68).

##### Retinopathy of prematurity (ROP)

The incidence of retinopathy of prematurity among infants born between 22 and 28 weeks gestational age remains 12.8% (69). Males are more likely to be screened for Retinopathy of Prematurity (ROP), and a higher percentage of male infants are treated for ROP as compared to female infants (55%, 95% CI: 52–58%) (43). Males are also at higher risk of having severe ROP (RR: 1.14, 95% CI: 1.07–1.22) (36, 44), suggesting that males are at higher risk of progression of ROP. This difference could, in part, be explained by the greater antioxidant capacity of female infants, both at the cellular level, wherein mitochondria from female cells produced fewer superoxide radicals than those of males (70), and at the metabolic level, wherein female infants had a more robust superoxide scavenging system, especially the glutathione pathway (71). Besides this, it is well established that preterm male infants are more likely to require intubation at birth, need higher and more prolonged respiratory support, and are at higher risk of other preterm morbidities such as sepsis and Necrotizing Enterocolitis (NEC) (36), all of which increase their risk of progression of ROP.

##### Hypoxic-ischemic-encephalopathy (HIE)

Hypoxic Ischemic Encephalopathy is a brain injury that is caused by inadequate blood supply to the brain as a result of a hypoxic-ischemic event that occurs during the perinatal period (72). The prevalence of HIE in term and late preterm infants in the United States remains approximately 1 per 1,000 live births (73). Animal studies have shown that males have worse outcomes associated with HIE than females. In a term rodent model of HIE, female mice were less likely to develop seizures and had a smaller infarct size as compared to male mice on day 3, along with lower inflammatory cell infiltrate in the brain as compared to male mice (39). This could be explained by recent studies suggesting that the primary apoptotic pathway in males, the Apoptosis-Inducing Factor (AIF) pathway, is more easily triggered by inflammatory stimuli than the caspase-3 pathway, which is predominant in females (74). In contrast to most recent human studies, Lee et al. (38) did show a significant but modest male predominance in a global metanalysis of the incidence of Intrapartum HIE (58.7%, 95% CI: 55.6–61.8). One possible explanation for the lack of any sex difference in treatment in more recent human studies could be inadequate sample size (41).

##### Therapeutic hypothermia

Post hoc analysis of the data from the Hypothermia trial (40) failed to show sexual dimorphism in the treatment effect of hypothermia in infants with moderate or severe neonatal encephalopathy (41). However, in animal studies conducted by Wood et al. (75), they found that hypothermia conferred a significantly more significant benefit in female animals as compared to male animals (median difference in area of brain loss between normothermia group and hypothermia group was 11.1% in females and 3.2% in males, *p* < 0.001). They postulated that this difference in the degree of benefit from hypothermia might be because hypothermia primarily suppresses the classical caspase-dependent apoptotic pathway, which is the dominant pathway of cell death in females, as compared to males, in whom the dominant pathway of cell death is caused by cellular depletion of NAD+ due to activation of PARP-1[poly(ADP-ribose)polymerase1] (76). Per Zhou et al. (77), “One reason for the lack of any obvious sex effects in humans and large animals may be that hypothermia suppresses a rather broad range of mechanisms of cell death (78), and so offers correspondingly broad protection between males and females.”

##### Neonatal abstinence syndrome (NAS)

The incidence of Neonatal Abstinence Syndrome in the United States increased from 4.0 per 1,000 live births in 2010 (95% CI: 3.3–4.7) to 7.3 per 1,000 live births in 2017 (95% CI: 6.8–7.7) (79). Multiple studies have looked into sex-related differences in adverse effects of in-utero exposure to opioids, with varying results. Unger et al. (80) carried out a secondary analysis of the data from the MOTHER trial (81), which was a double-blind, double-dummy, flexible dosing, randomized, controlled trial which compared buprenorphine with methadone for use in the comprehensive care of pregnant females with opioid use disorder. They found no specific sex-related differences in the variables concerning NAS course and treatment variables.

O'Connor et al. (31) conducted a retrospective cohort study of infants born to mothers who were on a buprenorphine treatment program and found that males had significantly higher mean peak NAS scores (10.04 vs. 7.98, *p* = 0.028) and were more likely to require pharmacological treatment for NAS (39.1% vs. 11.4%, *p* = 0.005). Similarly, Charles et al. (42) conducted a retrospective cohort study of mothers and infants enrolled in the Tennessee Medicaid Program and found that of the more than 100,000 infants enrolled in the study, male infants were more commonly diagnosed with NAS than females [adjusted odds ratio: 1.18 (95% confidence interval: 1.05–1.33)] and more likely to have NAS requiring treatment [adjusted odds ratio: 1.24 (95% confidence interval: 1.0401.47)]. However, of the 927 infants that were diagnosed with NAS, they found no sex-based differences in the severity of NAS. Jansson et al. (82) studied 65 infants born to mothers enrolled in an opioid addiction treatment program and found that males displayed significantly higher NAS scores each day than females (*p* < 0.05). They further found that although Males were not significantly more likely to be treated for NAS (81% vs. 69%, *p* ≫ 0.05) when they were treated, their treatment duration (13.4 vs. 9.0 days, *p* < 0.05) and their hospital stay was longer (15.9 vs. 12.0, *p* < 0.05). While no one coherent theory has been accepted for these results, animal studies have shown a similar heightened vulnerability of the male neonate to in-utero methadone exposure (83).

##### Long term

Preterm infants are at higher risk of Cerebral Palsy (CP), and this risk increases with decreasing gestational age (84). In a population-based cohort study of infants born at less than 27 weeks gestational age and surviving beyond one year, the lifetime prevalence of CP up to 6.5 years was 10.5% (85). Male sex has been identified in multiple studies as an independent risk factor for poor neurodevelopmental outcomes, including cerebral palsy (45–47), with Chounti et al. finding that males have a 30% higher incidence (95% CI: 11%–53% *p* = 0.002). An NRN database study (47) looking at neurodevelopmental outcomes in infants born at <28 weeks GA and with birth weight <1,000 grams at 18–22 months corrected age found increased odds for Neurodevelopmental Impairment in males as compared to females in the absence of severe IVH or PVL (OR, 95% CI for males vs. females: 1.79, 1.46–2.19). However, they further found that in the presence of severe IVH or PVL, there was no significant sex-related difference. They postulated that this sex difference is likely secondary to a central, biological difference between the sexes that is not currently quantifiable. These findings have been corroborated with large meta-analyses like the one done by Linsell et al. (86), which showed that in very preterm and very low birth weight infants, the male sex was an independent risk factor for global cognitive impairment at less than five years of age, however, in studies that assessed cognitive function at greater than five years of age, they did not find a sex-related difference. In terms of language development, multiple studies (87, 88) have shown a male disadvantage among preterm-born infants at 24 months of corrected age. Concerning the risk of Autism Spectrum Disorder (ASD), a study conducted by Allen et al. (89) followed 416 infants born prematurely (average GA 30.8 weeks, SD = 3.3) to the ages of 2–14 years (average age 4.2 years, SD = 2). Their results showed that among females, the risk of ASD was higher with lower gestational age, with the probability of ASD of 31% at 25 weeks vs. 0% at 32 weeks (*Wilcox approximately Z* = *2.7, p* < *0.*01). In preterm males, however, they found no significant difference in the gestational age of those who received a diagnosis of ASD (Mean = 31.7 weeks, SD = 3.2) vs. those who did not (Mean = 31.1 weeks, SD = 3.3, *p > 0.05*)*.* They theorized that this was due to a possible “two-hit hypothesis,” causing females to be at heightened risk of ASD mainly due to prematurity, while males remained at high risk for ASD even at near-term gestational age. Indeed, a *post hoc* analysis of the ELGAN study (90) found that in infants born between 23 and 27 weeks of gestational age, the male-to-female ratio of incidence of ASD increased with increasing gestational age from 2.1:1 to 4:1 (91).

These above findings of heightened male vulnerability to CNS insults and downstream consequences have been well documented in animal studies (39, 92). Theories explaining this sex-related heightened vulnerability include differential protective catecholamine response to in-utero hypoxia (93), chromosomal variants on X-chromosome (94), and immune dysregulation (39), to name a few.

#### Respiratory

##### Respiratory distress syndrome (RDS)

Respiratory Distress Syndrome (RDS) is one of the most common causes of morbidity and mortality in preterm neonates. The incidence of RDS is inversely proportionate to gestational age, with nearly 100% of infants between 22 and 24 weeks having RDS (95), 10% of male infants of European descent at 34 weeks, and down to 1% by 37 weeks (96).

It has been well established that males suffer a distinct disadvantage in terms of respiratory morbidities as compared to females. Male infants born preterm are at increased risk of RDS as compared to females (36) (RR = 1.090 (95% CI: 1.042–1.140, *p* < 0.001), as well as increased severity of RDS (48) with more males born <29 weeks gestational age requiring mechanical ventilation and surfactant therapy in the first 6 h of life as compared to females (60.8% males vs. 46.2% females, *p* = 0.026). Similar sexual dimorphism was seen concerning the risk of developing pneumothorax (36) (RR = 1.24, 95% CI: 1.104–1.393, *p* < 0.001), and Bronchopulmonary Dysplasia (BPD) (RR = 1.2, 95% CI: 1.091–1.319, *p* < 0.001). Similar differences were found in animal studies (97–99). The difference in early respiratory morbidity may be due to delayed maturation of male lungs, with females having the benefit of 17-beta estradiol and progesterone influence on surfactant protein expression (100), combined with the lack of inhibitory effect of androgens on surfactant production (101) and levels of tissue glucocorticoid receptor mRNA and protein (102). Fleisher et al. (103) found that the required 2:1 ratio of lecithin to sphingomyelin and the production of phosphatidylglycerol occurred more than a week earlier in female fetuses compared to males. Binet et al. (104) demonstrated that despite the use of exogenous surfactant and antenatal steroids, the female advantage for respiratory morbidities persisted.

##### Bronchopulmonary dysplasia (BPD)

The primary risk factor for developing Bronchopulmonary Dysplasia (BPD) is prematurity, with incidence varying widely between centers. This is likely due to differences in clinical management and varying definitions of BPD. According to data from the NICHD Neonatal Research Network (57), the rates of BPD have increased from 2009 to 2012, likely secondary to increased active resuscitation and survival of smaller and more immature infants. It may follow, given the need for higher and more prolonged respiratory support earlier in life in males, that they would be at higher risk of developing BPD. In a retrospective whole-population study consisting of nearly 12,000 infants born at less than 28 weeks gestational age, Dassios et al. (49) found that BPD was more common in males than in females among infants born at 24–27 weeks (*p* < 0.001) but not significantly different among males and females born at 22–23 weeks. These findings were corroborated by Farstad et al. (105). Dassios et al. postulated that the loss of male dominance in respiratory morbidity below 24 weeks may result from an inadequate protective effect of progesterone and 17-beta estradiol in female infants. Fulton et al. (106) analyzed the transcriptome of mesenchymal stem cells recovered from tracheal aspirates of 13 preterm infants and found that males who developed BPD expressed lower levels of specific genes involved in distal lung development. Hammond et al. (50) studied infants with established BPD. They found that there was no sex-related difference in rates of mechanical ventilation at 36 weeks (males 5.23%, females 3.6%), with OR = 1 for death or tracheostomy for male-to-female infants (95% CI: 0.7–1.5).

#### Cardiovascular

##### Blood pressure

Emery et al. (107) studied arterial blood pressure in infants with very low birth weights in the first 48 h of life. They found that male infants had significantly lower blood pressure as compared to female infants on the first day of life (mean of 42.4 mmHg in males vs. 45.6 mmHg in females, 95% CI: 05–5.6 mmHg, *p* < 0.05) and that this difference did not persist on the second day of life. More recently, Elsmen et al. (48) found in their study of infants <1,000 grams birth weight that males were at higher risk of requiring inotropic support in the first four days of life as compared to females (67.1% vs. 50.6%, *p* < 0.05), had lower mean arterial blood pressure in the first 12–24 h of life (25 vs. 28, *p* < 0.05), and were more likely to develop “resistant hypotension” defined as need for dobutamine or hydrocortisone in addition to volume and dopamine to treat hypotension (25.9 vs. 11.5, *p* < 0.05). Baik-Schneditz et al. (108) assessed cardiac output using electrical velocimetry in term neonates for 15 min after birth and found that males and females had comparable cardiac output at 5 and 10 min of life, but at 15 min of life, male infants had significantly higher cardiac out as compared to female infants (217 ml/kg/min vs. 178 ml/kg/min, *p* < 0.001).

##### Patent ductus arteriosus (PDA)

Van Westering-Kroon et al. (36) found no difference in rates of hypotension or Patent Ductus Arteriosus (PDA) between males and females in their meta-analysis of 41 studies. These findings were corroborated by a more recent meta-analysis by Borges-Lujan et al. (51), which included 146 studies and found no significant sex-related difference in the risk of developing any PDA (37 studies, RR: 1.03, 95% CI: 0.97–1.08) or risk of hemodynamically significant PDA (81 studies, RR: 1.00, 95% CI: 0.97–1.02). They also did not find a significant sex-related difference in the response rate to pharmacological treatment to PDA (45 studies, RR: 1.01, 95% CI: 0.98–1.04). There have been some studies with conflicting results, such as the one by Ahamed et al. (109), which found that the male gender was associated with a higher likelihood of successful PDA closure following Indomethacin treatment.

##### Long-term cardio-vascular system outcomes

Sheiner et al. (110) conducted a population-based cohort study of over 240,000 infants born between 1991 and 2013 and followed them up to the age of 18 years. They found that male sex (independent of birth weight or gestational age) was associated with a greater risk of pediatric cardiovascular morbidity (ARR: 1.37, 95% CI: 1.16–1.63, *p* < 0.001). They further found that male newborns exhibited a significantly greater incidence of total cardiovascular hospitalizations (log-rank *p* = 0.001), arrhythmia (log-rank *p* = 0.005), and heart failure (log-rank *p* = 0.023). In contrast, a study by Hovi et al. (111)found that adults who were born at a Very Low Birth Weight (VLBW) had higher systolic blood pressures than controls born at term (systolic 3.4 mmHg, 95% CI: 2.2–4.6), this difference was more marked in females (4.7 mmHg, 95% CI: 3.2–6.3) than in males (1.8 mmHg, 95% CI: 0.1–3.5). Other studies found no sex-related difference in the degree or incidence of hypertension in long-term studies of adults born preterm (112, 113).

#### SEPSIS

The rate of late-onset sepsis in Very Low Birth Weight infants has declined from 29.5% in 1995 to 2000 to 13% in 2013 to 2019 (114). Westering-Kroon et al. (36) found in their meta-analysis that male infants were at a significantly greater risk of late-onset sepsis as compared to female infants (R = 1.051, 95% CI: 1.026–1.077, *p* < 0.001), which was corroborated by Garfinkle et al. (115) (ARR for males = 1.04, 95% CI: 0.99–1.09) in their retrospective cohort study of Canadian infants. Similarly, Dutta et al. (52) found that male sex was an independent risk factor for early onset sepsis (RR = 2.7, 95% CI: 2–5). Indeed, even among infants with congenital CMV, there was found to be a sex-related difference, with females being more likely to have brain anomalies secondary to congenital CMV infection as compared to males (24% vs. 12%, *p* = 0.004) (116). Animal studies have indicated a sexual dimorphism in the immune response to infection (117). One of the explanations for this sex-based difference is the influence of sex hormones on immune function (118). For example, in murine T-cells, Araneo et al. (119) found that Dihydrotestosterone (DHT) exerts an immunosuppressive effect by reducing IL-4, IL-5, and IFN-γ production levels. Puder et al. (53) found that Estradiol inhibits the stimulatory effect of LPS (Lipopolysaccharide) on the Hypothalamic-Pituitary Axis (HPA) in adult females. Furthermore, Enomoto et al. (54)found that Estriol sensitizes Kupffer cells to LPS, which induces a strong response. The above is postulated (120) to be the primary reason females fare better against bacterial infections than males.

##### Necrotizing enterocolitis (NEC)

Necrotizing enterocolitis (NEC) is a disease that primarily affects preterm infants, with a worldwide incidence of 0.3–2.4 infants per 1,000 live births. It affects 2%–5% of all premature infants, with a mortality rate that ranges from 10% to 50%. Multiple studies have shown conflicting results regarding the presence of a sex-related difference in the risk of developing or dying from NEC. Shim et al. (121) conducted a retrospective observational study of Very Low Birth Weight infants in the Korean Neonatal Network. They found no significant sex-difference in the incidence of NEC at any gestational age. Carter et al. (121, 122) analyzed data from 134 infants less than 35 weeks gestational age at high risk of NEC due to birth weights of less than 1,500 grams or the need for mechanical ventilation at birth. Medical records of these infants were reviewed until the time of discharge. Medical NEC was defined as having pneumatosis intestinalis on x-ray and being treated with antibiotics for NEC for more than 48 h. Surgical NEC was defined as NEC requiring surgical intervention (peritoneal drain, exploratory laparotomy with diverting ostomy creation, primary anastomosis, intestinal resection, and stoma creation). Of the 134 infants, 24 developed symptoms that fit their criteria for NEC-15 males and nine females. The incidence of NEC in this study was 10% for males vs. 7% for females (*p* = 0.497). While their results did not reach statistical significance, they did show a trend towards increased susceptibility to NEC in males. These findings were corroborated by Ito et al. (123], who conducted a retrospective observational cohort study on Very Low Birth Weight Infants in the Neonatal Research Network of Japan between 2003 and 2012 with similar baseline demographics and rate of antenatal steroid exposure and found that among infants between the gestational ages of 23–25 weeks, NEC occurred at a significantly higher rate in male infants as compared to female infants (1.9 vs. 1.3, *p *< 0.001, OR = 1.469, 95% CI: 1.243–1.736). They postulated that the lack of sex-difference in older gestational ages was likely secondary to the rarity of NEC in infants born after 25 weeks gestational age. Similarly, Boghossian et al. (55) looked at single center data from 2006 to 2016 for 250,750 infants born between 22 and 29 weeks gestational age and found that males had a significantly higher rate of NEC as compared to females (10.1% vs. 8.4%, AOR = 1.2, 95% CI: 1.17–1.24), and remained constant throughout the study period. Since the etiopathogenesis of NEC is multifactorial and has not yet been clearly delineated, it is difficult to explain the role of sex in the risk of NEC.

Regarding outcomes following surgical NEC, Siahaan et al. (56) studied outcomes following a diagnosis of NEC in 52 infants. They found that female sex was associated with a 3-fold increase in risk of mortality. Similarly, Garg et al. (124) found that the female sex was associated with a higher risk of morbidity following surgical NEC, wherein morbidity was categorized as strictures, fistulas, wound dehiscence, surgical site infections with abscesses, any adhesions, and perforations.

#### Nutrition & growth

This topic has been extensively dealt with recently elsewhere, and we will summarize the findings in this review (13, 125). As discussed in this review, growth rates differ between male and female fetuses. These differences persist at birth and subsequently. It is important to note that the growth characteristics of preterm infants vary depending on their sex. Therefore, specific anthropometric standards are necessary for male and female preterm infants, such as Fenton's growth chart 2013, starting from 22 weeks of gestation onwards (126). To illustrate, at 24 weeks of gestation, the weight of male infants at the fiftieth percentile is 651 grams, compared to that of females at 606 grams. Additionally, male preterm infants have higher head circumference and length, which strongly indicates that they have higher growth rates.

#### Differences in body composition-males vs. females

The body composition of males and females differs significantly at birth. In term male infants, the body fat percentage is 9.57%, while in females, it is 11.54% (127). The InterGrowth 21st project revealed that males have a higher fat-free mass at 34 weeks of gestation (*p* < 0.001 (128). The body fat percentage is higher in females at 10.7% compared to 9.6% in males, and this difference increases over the first few months. It is worth noting that females, both preterm and term infants, have higher amounts of subcutaneous fat. A follow-up study conducted on preterm infants born less than 32 weeks showed that early postnatal weight gain is positively associated with BMI, waist circumference SD scores, fat mass, fat-free mass, and percentage body fat at 19 years of age (129). Long-term effects can result from changes in growth rate due to changes in body composition. The factors associated with differences in growth, growth velocity, and body composition among males and females are currently unknown.

#### Nutritional requirements—are they different?

Breast milk composition studies suggest possible sex differences in nutritional requirements. Both animals and humans produce sex-specific nutrient composition in breast milk. Human studies have shown that carbohydrate and caloric content may be higher in breast milk for male infants (125). According to the Add-Health study (130), breastfeeding had an impact on the growth of same-sex twins. Breastfed same-sex twins (either male or female) were found to be 1 inch taller and 12 pounds heavier than their opposite-sex counterparts during their adolescent years. On the other hand, same-sex twins who were never breastfed did not show any significant difference in height or weight compared to their opposite-sex counterparts. This finding suggests that breast milk composition may be tailored for each sex specifically to promote optimal growth.

A study conducted by Poindexter et al. (131) revealed that male preterm infants who received low early amino acid intake had a smaller head circumference at 18 months of age than those who received it later (47.7 ± 1.6 for the early and 47.2 ± 1.8 cm and late groups, respectively; *p* = .03). The odds ratio for males having head circumference less than the 10th percentile was 2.0 (95% CI: 1.0–4.0) and was 3.3 (95% CI: 1.4–7.7) for head circumference less than the 5th percentile. According to a recent study conducted in Europe (132), a higher intake of amino acids during the first week of life leads to higher weight gain in male infants during the first five weeks of life. Additionally, the study found that at 2 years of age, the mental developmental index (MDI) was higher in females, while the psychomotor developmental index was higher in males. Van den Akker et al. (133) in their only glucose vs. glucose with 2.4 g/kg/day of amino acids from birth, showed that VLBW males had 6.2 times [95% confidence interval (CI) 1.0–38.5] higher odds of achieving normal outcomes, i.e., without significant disability significantly (if amino acids were received from birth) more often than females. Females had a 10.1-point (95% CI: 18.6–1.6) lower MDI scores if amino acids were administered from birth onward. The studies suggest that providing the same early nutrition to premature infants of both sexes may result in different anthropometric or neurodevelopmental outcomes.

A study conducted in New Zealand (134) compared the nutrition provided to infants during the first week and the first month with their neurodevelopmental outcomes at the age of two years. The study found that although both males and females received similar nutrition, females had better survival rates without any neurodevelopmental impairments. They also noted that lipid intake during the first week was associated with better survival without neuro impairment in females. The limited data suggests that lipid provision may affect female preterm infants, while protein provision may impact males during early life. Robust prospective studies are needed to support this observation.

A retrospective review was conducted on extremely low birth weight (ELBW) infants born between 2014 and 16 at our level 4 neonatal intensive care unit (NICU) (*n* = 135). We investigated the impact of calories and protein on weight gain during the nutrition transition phase (TP) in extremely low birth weight (ELBW) infants, with a focus on sex differences. As expected, the calories and protein provided were similar in both sexes since NICU feeding guidelines were unisex. Therefore, equal amounts of volume, calories, and protein were given through nasogastric feeding. The entire group showed a significant correlation (*r* = 0.22, *p* = 0.026) between the intake of total calories and the change in weight percentiles. However, when analyzed by sex, the effect was only observed in females (*r* = 0.28, *p* = 0.015). Protein intake did not correlate with the change in weight percentile or sex (135).

*Significance*: Due to differences in their growth rate and body composition, preterm infants' nutritional requirements may vary according to sex. This is an essential consideration because preterm infants are incapable of regulating their intake through ad-lib feedings, and there are no sex-specific guidelines for providing nutrition.

Preterm infants born before 32 weeks of gestation lack an established sucking and swallowing reflex and an immature digestive system. As a result, provider-dependent nutrition is required for their growth and development. Oral feeds are attempted once these infants reach around 33 weeks of corrected gestational age, but significant respiratory support may make oral feeds unfeasible. This often results in prolonged enteral feeding via a nasogastric tube, with prescribed amounts of volume, calories, and protein to improve weight gain.

It is worth considering if preterm nutrition should be tailored based on sex, as suggested by postnatal nutrition studies. Despite having different growth trajectories, no studies have examined the potential differences in nutrition requirements between male and female preterm infants. Consequently, existing nutrition guidelines for preterm infants are not sex-specific and may not be optimal for both sexes.

## Discussion

An analysis of research literature has shown that infant sex plays a significant role in various aspects of neonatal outcomes, both before and after birth. A recent study suggests that the sex ratio is equal at conception, and overall mortality for females is higher during pregnancy (136). Although the National Institutes of Health (NIH) recommends including sex as a biological variable in research studies, researchers have not followed this recommendation strictly. Studies conducted on animals have revealed that therapeutic treatments may affect males and females differently. However, translating these findings into human studies has been slow to materialize. A better understanding of sex differences in response to drug therapies could help us achieve precision medicine. Therefore, more extensive and specific research studies are necessary to determine whether therapeutic hypothermia is beneficial for females (75) and whether caffeine is helpful for males. Similarly, we need precise research to confirm whether the early use of hydrocortisone prevents bronchopulmonary dysplasia (BPD) in females specifically (137) and whether prophylactic indomethacin reduces the incidence and severity of intraventricular hemorrhage (IVH) in males (37). Future research studies should also address whether a mother's own breast milk is more effective than donor breast milk in maintaining sex-specific body composition in premature infants.

## Part II. Effect of racial and social determinants of health on perinatal outcomes

Multiple factors determine health outcomes during pregnancy and the newborn period. Apart from the genetic, biological, and sex-based differences that influence fetal and neonatal outcomes, racial and social factors influence the health and well-being of developing humans. Race categorizes humans based on shared physical or social qualities into groups generally considered distinct within a given society (138). Social determinants of health (SDOH) are the non-medical factors that influence health outcomes. These factors can include a person's living conditions, access to healthy food, education, employment status, income level, and social support. Understanding these factors is essential in developing strategies to improve overall health outcomes in communities. The Centers for Disease Control and Prevention (CDC) has adopted the SDOH definition from the World Health Organization, which states that—SDOH are “the conditions in which people are born, grow, work, live, and age, and the wider set of forces and systems shaping the conditions of daily life. These forces and systems include economic policies and systems, development agendas, social norms, social policies, and political systems.” (139). Social Determinants of Health (SDOH) have been variously categorized based on society and living conditions' influence on human health and disease.

The intersection of race and SDOH is complex. Structural racism makes the interaction of race and social environment even more complicated (140). Although the effects of race and ethnicity are intertwined with SDOH, evidence exists that the effects of SDOH may be manifest independently (141–143). The goal of this review is to evaluate the current evidence for the role of Race and SDOH on maternal and neonatal outcomes.

### Effect of racial differences on neonatal outcomes

#### Introduction

Racial differences in neonatal outcomes explore the disparities in the health and survival of newborns from different racial and ethnic groups. It reflects the social and environmental factors that affect maternal and child health and the quality and equity of healthcare services. Studies have shown that there are significant differences in neonatal outcomes among non-Hispanic Black, non-Hispanic White, Hispanic, and other racial and ethnic groups in the United States and other countries. These differences are influenced by a complex interplay of biological, genetic, behavioral, cultural, and socio-economic factors and access to and quality prenatal and neonatal care. Understanding and addressing the causes and consequences of racial differences in neonatal outcomes is essential for improving the health and well-being of mothers and infants.

The infant mortality rate (IMR) has declined from 7.57 per 1,000 in 1995 to 5.89 per 1,000 births in 2015 (144). The IMR in the United States is higher than in Canada or England. Despite the decline in the IMR, the racial differences in infant mortality show huge differences between black and white infants (145). The infant mortality rate among white infants was 4.8 per 1,000 births in 2015 compared to 11.7 per 1,000 births among African American infants (146). Thus, in 2015, non-Hispanic black infants were 2.3 times more likely to die than white infants. The IMR for black infants remained at 10.55 compared to 4.36 for white infants in 2021. This high IMR among black infants is more than the other non-white races, such as 7.46 in American Indians and 4.76 in Hispanic infants (147). All the five major causes of IMR are 1.2–3.8 times higher in black infants than in white infants. The percentage of mothers who received first-trimester prenatal care is lower in non-Hispanic black mothers (68.4%) vs. non-Hispanic white mothers (82.8%). The percentage of mothers who received late or no prenatal care in 2020 was two times higher among non-Hispanic black mothers.

Annual trends in IMR were analyzed from 1999 to 2015. The average annual percent change for all-cause mortality by age and race in the USA was −1.99, −1.53, and −1.17 for black, Hispanic, and white infants, respectively, from 1999 to 2015 (148). A decrease in sudden infant death syndrome (SIDS) and congenital malformations was responsible for the decline among all races, followed by a decrease in short gestation/low birth weight among black individuals. However, the SIDS rate for black infants is still much higher at 80.52/100,000 births compared to 38.78/100,000 births in white infants in 2015. However, higher mortality rates were observed for unintentional suffocation and strangulation in bed among infants. Hence, better public education and SIDS campaigns may help reduce the IMR among all races, including black infants. Although mortality rates for children in the U.S. have improved significantly, they remain higher and are improving at a slower pace compared to Canada and England/Wales.

In a population-based retrospective cohort study in New York from 2010 to 2014 (6 years), the investigators analyzed the racial/ethnic differences in severe morbidities among 582,297 very preterm infants born at 24 weeks of gestation and later. The authors used a fetus-at-risk approach based on a collider stratification strategy (149). In the fetuses-at-risk analysis in this study, black infants have a higher risk of developing certain complications. Specifically, they have a 4.40 times higher rate of necrotizing enterocolitis (with a 95% confidence interval of 2.98–6.51), a 2.73 times higher rate of intraventricular hemorrhage (with a 95% confidence interval of 1.63–4.57), a 4.43 times higher rate of bronchopulmonary dysplasia (with a 95% confidence interval of 2.88–6.81), and a 2.98 times higher rate of retinopathy of prematurity (with a 95% confidence interval of 2.01–4.40). Hispanic infants had a nearly two times higher rate for all outcomes, and Asian infants had higher risk for retinopathy of prematurity alone (adjusted hazard ratio: 2.43; 95% CI: 1.43–4.11). Neonatal caregivers should be vigilant and ensure that all races receive the same evidence-based care to reduce biased outcome differences.

Vermont Oxford Network investigators analyzed 219,134 infants to understand if differences in outcomes between race and ethnicity changed over 12 years (2006–2017) in preterm infants born before 30 weeks gestation (150). The analysis included 40.6% white, 34.8% African American, 20.4% Hispanic, and 4.2% Asian American infants. Maternal hypertension increased in the groups but was highest in black mothers. The use of antenatal steroids showed an increase of 18.7% in black mothers vs. 13% in white and 21% in Hispanic mothers. In comparison to white infants, African American infants had a more rapid decrease in mortality, hypothermia, NEC, and LOS. On the other hand, Hispanic infants had a quicker decline in mortality, RDS, and pneumothorax. However, despite these improvements, by 2017, mortality and various health complications remained high, particularly among African American infants.

A study looked at the education level of the mothers to address whether socio-economic factors played a role in neonatal outcomes. The study used the U.S. vital statistics data sets, which included 2.2 million females (151). The researchers compared the maternal and neonatal outcomes of females with bachelor's degrees who delivered a normal live singleton baby between 24 and 40 weeks of pregnancy. Non-Hispanic black females had a higher risk of experiencing adverse maternal outcomes compared to non-Hispanic white females, while Hispanic females had a lower risk. Compared to non-Hispanic white females, non-Hispanic black females have a significantly higher risk of experiencing a negative maternal outcome (adjusted relative risk aRR: 1.20; 95% CI: 1.13–1.27). On the other hand, Hispanic females have a lower risk of experiencing a negative maternal outcome (aRR: 0.69; 95% CI: 0.64–0.74) when compared to non-Hispanic white females. The rate of adverse neonatal outcomes was 11.6 per 1,000 live births. The risk of adverse neonatal outcomes was significantly higher among neonates born to non-Hispanic black mothers (aRR: 1.25; 95% CI: 1.20–1.30) but lower among neonates born to Hispanic mothers (aRR: 0.71; 95% CI: 0.68–0.75), compared to neonates born to non-Hispanic white mothers. This risk also varied across gestational age. Thus, maternal education as a proxy of socio-economic status does not explain the racial differences in maternal and neonatal outcomes.

#### What do we know about racial differences and perinatal outcomes?

The purview of this article is broad, and hence, detailed discussion is beyond the scope of this article. We will summarize some of the findings that are currently available. Racial minorities have historically been limited to low-income neighborhoods (152) and received healthcare at lower-quality hospitals compared to the white population (153–155). Such suboptimal living conditions and access to healthcare can contribute to part of the racial differences in the health outcomes noted. Residential segregation in the United States is considered a manifestation of structural racism (156).

With its vast NICU database, Vermont Oxford Network attempted to study the extent of segregation (uneven distribution of racial/ethnic groups across NICUs) and inequality (concentration of racial or ethnic groups in lower-quality NICUs) (157). In their cohort of 117,982 very low-birthweight and very preterm infants, it was found that NICUs were segregated by race and ethnicity. They used Baby-MONITOR (Measure of Neonatal Intensive Care Outcomes Research), a hospital-level composite score of NICU quality based on nine infant-level measures. A higher score on the Baby-MONITOR indicates better quality care. Baby-MONITOR was tested in samples of California NICUs and is considered a strong indicator of quality-of-care delivery in the NICUs (158). This study looked at 743 hospital NICUs and found that Black, Hispanic, and Asian infants had NICU segregation indices of 0.50 (95% CI: 0.46–0.53), 0.58 (95% CI: 0.54–0.61), and 0.45 (95% CI: 0.40–0.50) respectively. This means that non-white infants went to different hospitals than white infants, showing significant segregation of minority patients The study found that Hispanic and Asian infants were treated at higher-quality NICUs than white infants, with NICU inequality indices of −0.10 (95% CI, −0.17 to −0.04) and −0.26 (95% CI, −0.32 to −0.19), respectively.

In contrast, the NICU inequality index for black infants was 0.07 (95% CI: 0.02–0.13), indicating that black infants were treated at lower-quality NICUs. The concentration of Hispanic parents in the regions with high-quality care hospitals may explain such differences to some extent. According to a regression model for Baby-MONITOR scores, a 10% increase in the proportion of black infants is associated with an estimated decrease of 0.05 in the Baby-MONITOR score. Similarly, a 10% increase in the proportion of Hispanic infants corresponds to an increase of 0.04 in the score, while a 10% increase in the proportion of Asian infants corresponds to a significant increase of 0.31 in the score. The findings suggest that there is a concerning correlation between the presence of a higher proportion of black infants in NICUs and lower-quality care. Even after adjusting for different regions, the results remained consistent. This implies that the concentration of black infants in lower-quality NICUs cannot be attributed to regional differences alone. These results underscore the need for further research and action to address the underlying causes of racial disparities in neonatal care.

The factors driving the segregation of minority infants into lower-quality NICUs are not fully comprehended. However, potential drivers include practices of residential segregation, systemic racism, poverty, and healthcare access-related factors like health insurance may play a role (159). Minority families often face limited options when seeking healthcare services. This is because their neighborhoods constrain their choice of healthcare facilities. The inability to access high-quality healthcare is closely linked to racial and economic segregation. For instance, a study conducted in New York found that females residing in neighborhoods that are racially and economically polarized are likely to give birth in hospitals located in similarly polarized neighborhoods (160).

A few studies examined hospital structure, such as nursing characteristics, to understand the racial differences in NICU outcomes (161). The authors studied two National Quality Forum (NQF) nurse-sensitive perinatal care standards, nosocomial infection and breast milk, which have long-term health implications for VLBW (162). High-black hospitals had greater rates of infection (18.4 percent vs. 14.3 percent; *p* < .001) and discharge without breast milk (63.4 percent vs. 43.0 percent; *p* < .001) compared to low-black hospitals. Both black and non-black infants had poorer outcome rates in the high-black hospitals. Understaffing was also higher in high-Black hospitals than in low-Black hospitals. However, in models controlling for nurse understaffing and the nursing practice environment, high-black hospital status was no longer significantly associated with either outcome. The results underscore the importance of nursing as a factor driving the disparities between these hospital types. This is further supported by the subsequent study, which evaluated missed nursing care in disproportionately black and non-black-serving hospitals (163). Missed nursing care was defined as necessary activities but left undone due to lack of time. It has been observed that there is a significant difference in the patient-to-nurse ratio between high-black hospitals and low-black hospitals. The nurses in high-black NICUs miss about 50 percent more nursing care as compared to those in low-black NICUs. The odds of missed care increase significantly with lower nurse staffing, while better practice environments decrease the odds. On average, nurses miss 1.23 care activities out of 12, most of which are in the planning/communication domain. It has also been observed that 44 percent of nurses miss one or more necessary nursing activities. This percentage is significantly higher in high-black NICUs (52%) as compared to low-black NICUs (38%). Therefore, hospitals should strive to ensure a better nurse-patient ratio to improve patient outcomes.

It is also reported that breast milk feeding rates are the lowest for black infants cared at high-black hospitals. However, when different NICUs were compared, black infants gained the most by being cared for in NICUs, with a higher percentage of white infants (161).

#### What needs to be done regarding race and perinatal outcomes

Increasing public awareness of racial and ethnic disparities in healthcare is crucial. We can achieve this by launching media campaigns and educational initiatives targeting healthcare consumers, payors, providers, and health systems administrators. Additionally, organizations responsible for training and licensing healthcare professionals should develop tailored programs to raise awareness of healthcare disparities among current and future providers. By increasing public and provider awareness, we can take the first step towards eliminating healthcare inequalities (164, 165).

According to Glazer et al.'s report, clear discrimination is not frequently observed. However, Black and Hispanic mothers often face disrespectful care and ineffective communication, which pose significant barriers to family engagement in infant care (166). This inadequate communication not only creates anxiety and stress for families but also impacts critical care processes in the NICU, such as skin-to-skin care and breastfeeding, which are essential for infant development. Hence, hospitals should invest in educating the clinical staff to prioritize communication and family engagement in patient care. A structured approach is proposed to address racial disparities by improving readiness, recognition, and response to the needs of minority families, reinforced by continuous learning within systems (167). However, these racial differences in neonatal outcomes are prevalent globally. A recent individual patient data meta-analysis of more than two million pregnancies from around the world showed that Black females are at increased risk of poor perinatal outcomes of neonatal death, stillbirth, preterm birth, and small-for-gestational-age babies than White females, even after adjusting for maternal characteristics (168). Hence, the involvement of the WHO may provide a better understanding of providing equitable care.

### Effect of SDOH on maternal and neonatal outcomes

#### Introduction

Fetal and neonatal health outcomes are determined not only by biological, genetic, or racial variables but also by social factors. As per the World Health Organization (WHO), “Social determinants of health (SDOH) are the nonmedical factors that influence health outcomes. They are the conditions in which people are born, grow, work, live, and age, and the wider set of forces and systems shaping the conditions of daily life. These forces and systems include economic policies and systems, development agendas, social norms, social policies, and political systems” (139).

The WHO has listed ten factors that can positively or negatively influence health (139). They are: “(1) Income and Social Protection; (2) Education; (3) Unemployment and Job Insecurity; (4) Working Life Conditions; (5) Food insecurity; (6) Housing, Basic amenities, and the Environment; (7) Early Childhood Development; (8) Social Inclusion and Non-discrimination; (9) Structural Conflict; and (10) Access to Affordable Health Services of decent quality”.

These factors influence all infants' conception, intra-uterine growth, birth, and postnatal health or disease. They also influence pregnancy status and pregnancy outcomes. Structural racism makes the interaction of race and social environment even more complicated (140). Although the effects of race and ethnicity are intertwined with SDOH, evidence exists that the effects of SDOH may be manifest independently (141–143). The goal of this review is to evaluate the current evidence for the role of SDOH on fetal and neonatal outcomes. For this review, the effect of a particular SDOH factor on pregnancy, fetus, and newborn infants after birth will be evaluated based on the ten distinct SDOH categories listed under the WHO definition (139). Only findings from clinical and epidemiologic studies will be included, and non-human studies will be excluded.

Factors within the SDOH are interrelated and can affect pregnancy outcomes through complex interactions (169, 170). Pre-pregnancy health and health behaviors, such as hypertension and lack of physical activity, can increase the risk for maternal morbidity and mortality. These factors are influenced by the availability of safe places to exercise and access to affordable, nutritious food (171). The financial insecurity resulting from SDOH often compromises the physical and mental health of females (172). Additional studies of maternal health emphasize the influence of racism on stress, health, and well-being (173).

SDOH also has an effect on neonatal outcomes at multiple levels (174). Temporal trends for outcome changes based on SDOH in the U.S. have not been reassuring. The infant mortality discrepancy between the high and low Socioeconomic Status (SES) groups, as shown in a study using US Vital Statistics The data indicates a significant difference in post-neonatal and infant mortality rates between individuals belonging to lower and higher socio-economic status groups. In 1985–89, the neonatal mortality rate was 36% higher in the most deprived group as compared to the least deprived group. However, the gap increased to 43% higher neonatal mortality rate in the most deprived group in 1995–2000 (175).

Evaluating the effect of individual items within the SDOH construct is very near impossible. However, individual studies have tried to focus on one or more factors as the primary drivers of outcomes. In this review, we will classify the primary factor within one of the ten items of SDOH and evaluate the current evidence of their effect.

#### Specific SDOH items and maternal/neonatal outcomes

1. Income, insurance, or social protection

The wealth gap between white families and black families in the U.S. is vast, with the median white family holding almost ten times more wealth than the median black family. One of the primary factors contributing to this disparity is historical redlining, a discriminatory practice that restricted access to financing and economic opportunities, leading to the development of highly segregated communities across the country. Generally, communities with lower levels of wealth and income, and higher levels of poverty, are at a greater risk of suffering from morbidity and mortality (176).

A large retrospective cross-sectional study using the U.S. multicenter Kid's Inpatient Database evaluated newborn infants diagnosed with sepsis and compared mortality with maternal SDOH, including insurance coverage, household income, and race. There was increased mortality (3.26 times higher) among those with self-pay when compared with privately insured families. Families with low household income had 1.19 times the odds of mortality compared to those with higher household incomes families (177). It has been found that babies born to low-income families have a higher rate of low birth weight, preterm birth, infant mortality, and developmental delays compared to those born to higher-income families (178–181). A study based on the Niday Perinatal Database from Ontario also showed that lower ranges of neighborhood income were associated with increased risks of stillbirths. In live-born infants, there was a higher incidence of small for gestational age babies, low birth weight, and preterm birth (182).

The relationship between socio-economic status and neonatal outcomes is complex. In one large population study conducted in Europe, there were no associations between income levels alone and neonatal outcomes of prematurity or mortality. However, when there is a combination of income level with one more SDOH-related factor, the risk for prematurity increases significantly (183).

2. Education and literacy

Education interplays with health and other social factors in many ways. Higher education is associated with a higher socio-economic status (SES). However, Education is not always a proxy for SES (184). Lower education level was highly correlated with late prenatal care and LBW irrespective of the socio-economic status of the families (184). One reason for this may be because education improves the ability of parents to access and understand health information and services for themselves and their babies. Other comprehensive reviews on the subject have also concluded that babies born to mothers with low levels of education are more likely to have poor health outcomes than those born to mothers with higher levels of education (179, 180).

Analysis of U.S. National Vital Statistics System data linked to county-level socio-economic data consisting of education indicators, among others, showed a greater mortality associated with lower maternal education. The discrepancy worsened with each lesser year of schooling. It was, however, difficult to tease out the independent effect of education level from other factors such as social conditions, smoking during pregnancy, and availability of healthcare services (175). A three-generation study noted that a higher level of grandmother's education was associated with higher birth weight in the grand offspring, especially if the mother's education level was not very high (181).

3. Unemployment and job insecurity

While strenuous work during pregnancy may be harmful to pregnant females's health, being unemployed and the associated burdens of its consequences probably also have an adverse effect on pregnant mothers and their babies. Data from Texas shows that unemployment is associated with lower birthweights and higher infant mortality rates than employment (185). The effect was especially notable when unemployment preceded market work (185).

The effect of one or both partner employment is also significant. Four groups were defined and analyzed in an Australian study to evaluate employment status based on one or both unemployed partners (186). “The groups were: *Group 1* females unemployed, partners not unemployed. *Group 2* females not unemployed with unemployed partners. *Group 3* comprised females and partners who were both unemployed (186). In *Group 4*, neither partner was unemployed”. Although unemployment of any of the partners was associated with a higher risk of LBW and PTB, the association was less robust when other health factors, such as smoking, were included in the analysis (186). According to a report on unemployment that took into account either one or both partners being unemployed, it was found that unemployed females were more likely to have infants who were small-for-gestational-age (SGA), with an odds ratio (OR) of 1.26 (95% CI: 1.12–1.42). In families where both parents were unemployed, the risk of SGA was even higher, with an OR of 1.43 (95% CI: 1.18–1.73) (187).

The effect of unemployment may also vary with the safety net provided by unemployment benefits in different countries. A study during the Great Recession evaluated the effect of unemployment variations and birth outcomes in Britain (188). In this study, they found that unemployment most adversely affects babies (LBW and PTB) conceived in the average to the lowest socio-economic areas. In contrast, the opposite is true for the ones conceived in the wealthiest areas. Their data also shows that average to lowest SES babies are most damaged by recessions (188). A study of births in Spain found that females from regions with high unemployment rates had double the risk of stillbirth (adjusted OR: 2.60; 95% CI: 2.08–3.21) (189). A birth and death linked database from the Netherlands was studied, and it was found that Perinatal mortality was independently associated with the father's and mother's employment status (190). In a longitudinal study of families from the U.K. According to the ‘Understanding Study,’ pregnant females who experienced job loss, whether it was their own or their partner's, had a higher risk of pregnancy loss. This increased risk persisted even after considering socio-economic and partnership-related factors. The odds ratio for this risk was 1.81, with a 95% confidence interval of 1.20–2.73 (191).

Countries with a robust unemployment benefits program tend to have a lower adverse effect on unemployment and job security. This suggests that the issue of unemployment and job insecurity affecting pregnancy and newborns is very complex and nuanced and needs further evaluation.

4. Working life conditions

The effect of work-life stress and work hours during pregnancy is expected to affect the fetus and newborn. There is probably a threshold effect with adverse outcomes above a particular threshold. The Amsterdam Born Children and Their Development study found a significant association between reduced birthweight and extended work week of more than 32 h (mean decrease of 43 g) and high job strain (mean decrease of 72 g) (192). Longer work week >32 h (mean birthweight decrease of 43 g) and high job strain (mean birthweight decrease of 72 g) were significantly associated with birth weight (192). In another study, working >50 h/wk [odds ratio (OR) = 1.59], standing more than seven hr/d (OR = 1.40), and no antenatal leave (OR = 1.55) were associated with an increased risk of IUGR (193).

Job hazards, especially with manual work during pregnancy, may have an adverse impact on the mother and the fetus. In one study, job hazards contributed to very low birth weight and extremely preterm births, and physical demands of work contributed to low birth weight and all preterm births (194). In a cross-sectional, population-based study from Norway, it was shown that the adverse effects (prematurity and LBW) of strenuous work during pregnancy were primarily seen in nulliparous females (195).

Job stress may be another factor of influence. A population-based study from Denmark showed an increased risk of spontaneous abortion [OR = 1.28, 95%, (CI) 1.05–1.57] for females with high job stress (196). Even after accounting for potential bias, they found worse neonatal outcomes for congenital malformation, 1.23 (95% CI: 0.93–1.63); prematurity, 1.03 (95% CI: 0.77–1.39); small for gestational age, 1.08 (95% CI: 0.83–1.40); and stillbirth/death within the first year of life, 1.42 (95% CI: 0.90–2.24) (196).

Night shifts at work are another contributor to pregnancy-related stress on the mother and fetus. In a Danish National Birth Cohort study, researchers found that fixed night work was associated with fetal loss (OR = 1.85, 95% CI = 1.00–3.42). However, as measured in this study, job stress was not associated with fetal loss (197).

5. Food insecurity

Food insecurity, especially during pregnancy, has an impact on the mother and the unborn fetus. In a systematic review of published studies, it was found that food -insecurity was associated with higher maternal stress and higher neonatal mortality, especially in studies from Africa (198). In contrast, one study from Malawi failed to show a relationship between food insecurity and adverse pregnancy outcomes, although the overall status of the population may have confounded this issue (199). In one retrospective mother-infant dyad study from the U.S., the risk of prematurity was three times higher (95% CI: 1.0–8.9, *P* = .05) in pregnant mothers who experienced food insecurity (200).

Infant sex, among other factors, may modify food insecurity risk (Based on PRAMS data of live births from 11 states during 2009–2017.) (201). They found that food-insecure mothers had a significantly increased risk of delivering a low-birthweight baby. Other covariates could account for the association among male infants, but the magnitude of risk remained high in female infants despite adjusting for covariates (adjusted OR: 1.13; 95% CI: 0.94, 1.35) (201).

Evidence for this factor also comes from the effects of proper correction of food insecurity in low and middle-income countries where balanced protein-calorie supplementation programs have shown a decrease in stillbirth rates with a Risk Ratio of 0.60% and 95% CI: 0.39–0.94 (202). More studies and systematic reviews are currently in progress to fully explore this relationship (203).

6. Housing, neighborhoods, basic amenities, and the environment

The neighborhood environment influences the *in-utero* and *ex-utero* health of the baby. Neighborhood and community factors are critical influences on the quality of health care and social support for babies and their families (179, 180). Females from disadvantaged neighborhoods have a 27% higher risk of prematurity and 11% higher risk for low-birth-weight babies (204). Babies born to families living in neighborhoods with high levels of deprivation, crime, violence, pollution, lack of resources, and opportunities are more likely to experience poor health outcomes than those born in families that live in more advantaged neighborhoods (205). Using a composite measure of neighborhood influences—the Childhood Opportunity Index (COI) (145), Shanahan et al. showed a significant relationship between poor COI and poor life expectancy at birth (205). Specific neonatal morbidity involving intraventricular hemorrhage (IVH) in the brain is higher in communities that have residential racial segregation (RSS) with compounding effects of race and residence (180, 206).

The actual condition of the house itself may be an additional risk factor. In a study of housing conditions of indigenous people in Canada, the need for major household repairs was associated with an increased risk of infant death (aRR = 1.69, 95% CI: 1.00–2.85) (207). In one particular indigenous group, the First Nations, household crowding was also associated with an increased risk of infant mortality (aRR = 1.57, 95% CI: 0.97–2.53) (207).

Neighborhoods with exposure to environmental pollutants are known to contribute to maternal morbidities such as preeclampsia and placental abruption, which in turn can raise the risk of maternal mortality (208, 209). Exposure to housing renovation during the periconceptional period increases the risk of Congenital Heart Disease (adjusted OR: 1.77, 95% CI: 1.34, 2.33) (210).

The stability of a household plays a role in the evaluation of housing and its effects on health. In a systematic review of housing instability and its impact on perinatal outcomes, the reviewers found that housing instability and homelessness while pregnant were considerably associated with preterm birth and delivery complications in the mother (211). The babies born to these mothers had worse outcomes, such as low birth weight and neonatal intensive care unit admission (211).

7. Early childhood development

An area of great concern is that childhood adversity may have health consequences for the current and future generations. In one study, a mother's childhood economic hardship (assessed by a questionnaire) was associated with multiple adverse neonatal outcomes, even after adjusting for other confounders such as demographics, maternal education, and obstetrical conditions. Females raised in disadvantaged conditions had higher prematurity rates. Their babies had lower birth weights, were more likely to be small for gestational age, and more extended hospital stays (212). The Childhood Opportunity Index (COI) is a validated measure of early childhood influences on health. Females from lower COI areas had higher adverse pregnancy outcomes and had newborns with lower birth weight, birth length, and head circumference (213). An interesting association has been discovered between a woman's childhood exposure to educated parents and the birth weight of her offspring. If the grandmother is educated and even if her daughter is not as well educated, the progeny of that child is protected from being LBW (181).

Another type of childhood adversity is sexual abuse. Females exposed to childhood sexual abuse (CSA) had more complicated pregnancies (41.2%/19.4%; OR: 2.91, CI: 1.64–5.17). They also had more complications such as premature contractions (38.8%/20%; OR: 2.54 CI: 1.43–4.51), cervical insufficiency (25.9%/9.4%; OR: 3.36, CI: 1.65–6.82), and premature birth (18.8%/8.2%; OR: 2.58, CI: 1.19–5.59) (214).

When adverse childhood experiences (ACE) were semi-quantitatively analyzed for their effect on later pregnancy-related outcomes, a study from Wisconsin reported that cumulative ACE scores were associated with an increased pregnancy loss (OR = 1.12; 95% CI = 1.08–1.17), preterm birth (OR = 1.07; 95% CI = 1.01–1.12), and low birth weight (OR = 1.08; 95% CI = 1.03–1.15) (215). There is probably a threshold effect of the quantity of adverse childhood experiences (ACE). In one report, mothers exposed to 4 or more ACEs had a 3.74 times risk for low birth weight (0.050 vs. 0.187) and a 1.74 times greater risk for prematurity (0.085 vs. 0.148) than those whose mothers reported no ACE exposure (216). The biological basis for adverse early childhood events leading to adverse maternal and fetal outcomes later in life may be explained by epigenetic changes and DNA methylation (217).

8. Social inclusion and non-discrimination

Evaluation of social inclusion or discrimination is subjective. However, several objective methods exist for categorizing this variable in evaluating mother and newborn outcomes. Based on the cohort identified in the Black Females' Health Study, further study was done among Black people who experienced discrimination or other forms of racism. The results of the study indicated that females who reported unfair treatment at their workplace had an adjusted odds ratio (O.R.) of 1.3 [with a 95% confidence interval (CI) of 1.1–1.6] for preterm birth. Similarly, females who reported experiencing fear from others at least once a week had an adjusted O.R. of 1.4 (with a 95% CI of 1.0–1.9) for preterm birth.

A report from the subjects enrolled in the CARDIA study evaluated the preterm and LBW outcomes among black persons and white persons based on their perceived level of discrimination. It was found that self-reported experiences of racial discrimination were associated with higher odds of preterm Adjusted OR: 1.11 (95% CI = 0.51, 2.41 and low birthweight Adjusted OR: 2.43 (95% CI = 0.79, 7.42) deliveries (218).

Discrimination interfaces not only with racism but also with depressive symptoms and stress. A study quantified racism based on a score and reported its interaction with other factors. They found that high Racism scores were associated with a higher risk of preterm birth in three subgroups where depressive symptoms and stress modulated the effect (219). The effect of discrimination varies based on ethnicity. A cross-sectional study was done with data from the Community Child Health Research Network multisite cohort of subjects, showing that African American and Latina females with the highest tertile of discrimination had a higher prevalence of preterm birth adjusted hazard ratio (aHR) = 1.5 (95% CI; 0.7–3.1) and 3.6 (95% CI: 0.9–14.4), respectively (220). Discrimination based on immigration status was shown to affect pregnancy outcomes in Turkish immigrants in Germany. Within the subsample of Turkish immigrant females, perceived discrimination was related to a significantly higher PTB risk [OR: 4.91, 95% CI (1.76–15.06)] (221). In a systematic review of studies on discrimination and its effects on adverse obstetric outcomes, it was reported that perceived racism was associated with poor obstetric outcomes (222).

A decrease in racial discrimination in black persons can occur with a reduction of inequity in education. In such instances, it has been shown that there was a 10% decrease in black infant mortality when educational inequity with white persons was eliminated (223).

9. Structural conflict

Although race and ethnicity are categories that independently influence health and outcomes, they are also determinants of social conflict. Babies born to racial and ethnic minorities, especially Black, Hispanic, and American Indian/Alaska Native families, face higher risks of preterm birth, low birth weight, infant mortality, and other health problems than white babies (179, 180). These disparities are partly explained by the effects of centuries of structural racism, discrimination, and historical trauma on the health and well-being of these communities (169, 179, 180). Because of the legacy of redlining and sustained housing segregation, pregnant and birthing people of color are more likely compared to white pregnant or birthing people to reside in communities with higher rates of instability, interpersonal violence, crime, and over-policing.

Conflict and an environment of violence are associated with higher maternal mortality (224). Intimate Partner Violence (IPV), specifically, has been shown to affect maternal health adversely (e.g., poor prenatal care, poor nutrition, or inadequate weight gain), as well as adverse neonatal outcomes (low birth weight, preterm birth, and intrauterine growth restriction) and maternal and neonatal mortality (225). Mass incarceration is correlated with higher maternal mortality and higher rates of prematurity (226).

10. Access to affordable health services of decent quality

Access to health care depends on income, insurance, and housing location. Redlining and other structural factors have led to black persons living in poorer neighborhoods with access to hospitals that may also provide poorer care. In a study examining the relationship between Infant Mortality (I.M.) and the Maternal Health Care Services Access Index (MHCI) in Nigeria, it was noted that The I.M. rate reduced from 119 per 1,000 live births among females whose MHCI score was poorest to 50 per 1,000 live births among those with the best MHCI access score (227).

In a study using data from the Nationwide Inpatient Sample of the Healthcare Cost and Utilization Project, a federal-state-industry partnership that the Agency sponsors for Healthcare Research and Quality, it was found that deliveries in high and medium black-serving hospitals had higher rates of severe maternal morbidity rates compared with those in low black-serving hospitals in adjusted analyses (17.3 and 16.5 vs. 13.5 per 1,000 deliveries, respectively; *p* < .001) (228). In this case, there was an interaction of race and neighborhood, with black females delivering at high black-serving hospitals having the highest risk of poor outcomes (228). However, another study based on the State Inpatients Database for New York showed that for their effects on maternal morbidity and mortality, patient-related factors such as access to care were more significant than the hospital or neighborhood-related factors (229).

The urban-rural gap in health access also affects infant outcomes. A California Perinatal Quality Collaborative study reported that major morbidity in VLBW survivors decreased with increasing rurality, and the relationship remained significant for small rural/isolated areas (OR: 0.79, *p* = 0.03) (230).

Access to health has a high potential of being correctable. Efforts to improve access have already shown an improvement in health outcomes. Availability of insurance coverage increased under the Affordable Care Act, and thus, an improvement in access led to a reduction in maternal mortality and morbidity (231).

## Discussion

It is clear from a review of the literature that race and social determinants of the health of families have a significant impact on various aspects of neonatal outcomes. Higher infant and neonatal mortality in black infants may, in part, be related to inherent biological differences. However, differences in neonatal and perinatal outcomes based on race and social determinants of health have a complex relationship. Disadvantaged races appear to get care in underprivileged areas where hospital staffing and quality indicators are poorer, and it is not surprising that they are associated with poorer health outcomes. The continuing perpetuation of this vicious cycle may be one of the explanations for the persistent temporal trends of poorer outcomes in blacks without a significant narrowing of the racial divide.

Among the various contributors to the social determinants of health, many are interrelated and co-related with each other and other racial and ethnic influences, creating cumulative effects on babies and their families. In the U.S., SDOH disproportionately affects people of color, owing to systemic racism and policies dating back a few centuries that have been discriminatory in their design and implementation. These policies have resulted in significant racial disparities in healthcare access, education, food security, safe housing, employment, and ultimately health outcomes. Addressing these factors requires comprehensive and coordinated policies and interventions that promote health equity and social justice for all babies (179, 180). Recognizing this, there has been an increase in programs addressing SDOH as part of the healthcare spending in the U.S., with about $ 2.5 billion spent between 2017 and 2019 (232). There have been attempts to synthesize the interplay of different SDOH items and formulate SDOH patterns, which can then be analyzed within a defined group—SDOH pattern one from affluent communities and lowest social vulnerabilities; SDOH pattern 2—from high stigma environment and high level of implicit bias; SDOH pattern three from highly deprived socio-economic environments characterized by low income and poverty; and lastly SDOH pattern four from high crime and disruptive environments with lowest levels of support and highest levels of disruption (233). In a study using these patterns, physical and mental outcomes in later childhood were highly correlated with the SDOH patterns (233). Furthermore, new data collection and analysis tools using data mining from EMRs could incorporate SDOH to help anticipate and manage population health (234).

Achieving broad and ambitious goals requires specific and achievable objectives. National attention to SDOH and its relation to maternal and newborn outcomes has helped develop the “Healthy People 2030” vision. Healthy People 2030 sets data-driven national objectives to improve health and well-being over the next decade. Healthy People 2030's overarching goals are to “(i) Eliminate health disparities, achieve health equity, and attain health literacy to improve the health and well-being of all. (ii) Create social, physical, and economic environments that promote attaining the full potential for health and well-being for all. (iii) Promote healthy development, healthy behaviors, and well-being across all life stages”. The future of health care is not only about managing disease but also about understanding the personal factors (race, ethnicity, and sex) and social determinants of health and developing strategies to promote their beneficial effects and prevent their adverse effects.
